# 25OHD analogues and vacuum blood collection tubes dramatically affect the accuracy of automated immunoassays

**DOI:** 10.1038/srep14636

**Published:** 2015-09-30

**Authors:** Songlin Yu, Xinqi Cheng, Huiling Fang, Ruiping Zhang, Jianhua Han, Xuzhen Qin, Qian Cheng, Wei Su, Li’an Hou, Liangyu Xia, Ling Qiu

**Affiliations:** 1 Department of Clinical Laboratory, Peking Union Medical College Hospital, Chinese Academy of Medical Sciences, Beijing 100730, China; 2 Department of Clinical Laboratory, China-Japan Hospital, Beijing 100029, China

## Abstract

Variations in vitamin D quantification methods are large, and influences of vitamin D analogues and blood collection methods have not been systematically examined. We evaluated the effects of vitamin D analogues 25OHD_2_ and 3-epi 25OHD_3_ and blood collection methods on vitamin D measurement, using five immunoassay systems and liquid chromatography-tandem mass spectrometry (LC-MS/MS). Serum samples (332) were selected from routine vitamin D assay requests, including samples with or without 25OHD_2_ or 3-epi 25OHD_3_, and analysed using various immunoassay systems. In samples with no 25OHD_2_ or 3-epi 25OHD_3_, all immunoassays correlated well with LC-MS/MS. However, the Siemens system produced a large positive mean bias of 12.5 ng/mL and a poor Kappa value when using tubes with clot activator and gel separator. When 25OHD_2_ or 3-epi 25OHD_3_ was present, correlations and clinical agreement decreased for all immunoassays. Serum 25OHD in VACUETTE tubes with gel and clot activator, as measured by the Siemens system, produced significantly higher values than did samples collected in VACUETTE tubes with no additives. Bias decreased and clinical agreement improved significantly when using tubes with no additives. In conclusion, most automated immunoassays showed acceptable correlation and agreement with LC-MS/MS; however, 25OHD analogues and blood collection tubes dramatically affected accuracy.

Recent evidence for the association between vitamin D and bone disease^1^, diabetes^2^, autoimmune diseases^3^, cardiovascular diseases^4^, and cancer^5^, coupled with the recognition that vitamin D deficiency is common, has led to a massive rise in vitamin D testing worldwide^1^,^6^,^7^. For example, at the Mayo clinic, tests for vitamin D deficiency have increased by 80%–90% per year^6^, and in our laboratory, the number of tests given have doubled within the last two years. Because of this increased workload, automated immunoassays have recently been developed. However, there is large variation among methods used to test for vitamin D levels^6^,^8^,^9^.

Circulating 25 hydroxyvitamin D (25OHD) is the predominant form of vitamin D and is generally considered to be the most reliable biomarker of serum vitamin D concentration^10^. 25OHD_3_ and 25OHD_2_ are the two main types of 25OHD: 25OHD_3_ is endogenously produced in animals in skin in response to sun exposure and may be obtained in the diet through 25OHD_3_-containing supplements. 25OHD_2_ is found in fungi. Either 25OHD_2_ or D_3_ may be used in supplements or as additives to foods such as dairy products^1^. However, 3-epi 25OHD_3_, an epimer of 25OHD_3_, has recently been identified. While the bioactivity of this analogue is still unclear^11^,^12^, 3-epi 25OHD_3_ has been shown to cause cross-reactivity with 25OHD_3_ in immunoassays, leading to overestimation of 25OHD levels.

Various automated immunoassays have been developed for detection of 25OHD. However, the variation between laboratories has been reported to be as high as 38%^6^,^8^,^13^. Additionally, the definition of vitamin D deficiency is still controversial, with large variations among methods contributing to the controversy. Such large variations are also expected to be problematic for clinical diagnostic applications using the same cut-off for vitamin D deficiency for different methods. Therefore, the clinical uniformity of the different methods should be evaluated.

Isotope-dilution liquid chromatography-tandem mass spectrometry (LC-MS/MS) is considered the gold standard and reference method for 25OHD testing^14^. However, the most routinely used LC-MS/MS approaches and those that formed the basis for comparing methods have not included the separation of 3-epi 25OHD_3_ from 25OHD_3_ because it would require time-consuming analysis via chromatography, which would decrease detection efficiency; only in isolated studies has 3-epi 25OHD_3_ been measured using LC-MS/MS^14^,^15^.

Several studies have compared methods for vitamin D detection in recent years; however, whether such methods meet performance standards is controversial. In 2012, Farrell *et al.*^8^ compared the performance of five automated immunoassays with LC-MS/MS and concluded that the Roche system did not meet the minimum performance goals, while Ajuria-Morentin *et al.*^9^ reported that the Siemens system had the largest bias in comparison to LC-MS/MS, without a clear explanation for this bias. Both vitamin D_2_ and vitamin D_3_ supplements are heavily used in China and the United States of America (USA), the identification of significant concentrations of circulating 25OHD_2_ and 3-epi 25OHD_3_^11^ has led some manufacturers, such as Roche, to update their products to improve detection and quantification efficiency for distinct analogues. However, these novel immunoassays may be limited by antibody cross-reactivity and non-equimolar recognition of 25OHD_2_ and 25OHD_3_. Moreover, no studies have evaluated the effects of 25OHD_2_ and 3-epi 25OHD_3_ on the performance of the latest generation of 25OHD immunoassay systems. As an additional complication of accurate 25OHD measurement, the use of vacuum blood collection tubes is not standardized, and different collection tubes may affect the accuracy of immunoassays.

In this study, we compared five automated immunoassays including Roche Cobas E601 (Roche Diagnostics (Shanghai) Ltd., Basel, Switzerland), Siemens ADVIA Centaur XP (Siemens Healthcare Diagnostics (Shanghai) Co., Walpole, USA), DiaSorin Liaison XL (DiaSorin, Saluggia, Italy), Abbott Architect I4000 (Abbott Diagnostics, Deerfield, IL, USA), and IDS-iSYS (IDS France, Pouilly en Auxois, France) with a reference LC-MS/MS method to evaluate the effects of 25OHD_2_ and 3-epi 25OHD_3_ on the accuracy of the five automated immunoassays and to determine the influence of additives in vacuum blood collection tubes on the detection of 25OHD.

## Materials and Methods

### Study design

From April to June 2014, 332 serum samples from 106 males and 226 females, ranging from 6 months to 93 years of age (mean ± SD; 46 ± 22 years old), were collected from routine vitamin D assay requests. Samples were collected using VACUETTE 4-mL tubes with gel and clot activator (REF: 454067; Greiner Bio-one, Kremsmunster, Austria), including 166 serum samples containing 25OHD_3_ (4.3–57.4 ng/mL), 111 serum samples containing 25OHD_2_ (2.5–78.4 ng/mL), 31 serum samples containing 3-epi 25OHD_3_ (2.2–8.8 ng/mL), and 24 samples containing both 25OHD_2_ (3.2–18.8 ng/mL) and 3-epi 25OHD_3_ (2.2–5.4 ng/mL), as well as 25OHD_3_. At the time of this study, LC-MS/MS was used in our laboratory and served to select the samples for this study. Serum samples were divided into six aliquots and stored at −80 °C to ensure stability until analysis^16^,^17^.

For investigation of the possible effects of additives in vacuum blood collection tubes on 25OHD measurement, 77 healthy volunteers were recruited, and fasting blood samples were collected from each individual by venipuncture into VACUETTE 4-mL additive-free tubes (REF: 454001; Greiner Bio-one); samples were then centrifuged within 2 h (1200 × *g*, 10 min) and immediately analysed using automated immunoassay systems and LC-MS/MS.

We used LC-MS/MS to measure 25OHD and to evaluate the five automated chemiluminescence immunoassay systems. In addition, three serum pools were prepared for the assessment of assay precision. The LC-MS/MS method demonstrated mean 25OHD concentrations of 6.2, 16.0, and 22.1 ng/mL, respectively, for the three pools, with concentrations of 25OHD_2_ and 3-epi 25OHD_3_ less than 2.5 ng/mL. Multiple aliquots from the three pools were prepared and stored at −80 °C. For 5 consecutive days, a freshly thawed aliquot of each pool was assayed four times using all methods.

The study had been reviewed and approved by the Ethics Committee of Peking Union Medical College Hospital, and the experiments were carried out in accordance with the approved guidelines. All studied individuals were informed in writing of the intended use of their samples and each provided written consent.

### Measurement of 25OHD by LC-MS/MS and immunoassays

LC-MS/MS was performed using a Waters ACQUITY UPLC system (Waters Corporation, Milford, MA, USA) in tandem with an AB Sciex 4000 QTrap system (Sciex Applied Biosystems, Foster City, CA, USA). The protocol for sample preparation was as follows. Serum samples, calibrators, and controls were treated with 0.1 mM sodium hydroxide and precipitated with 1 mM zinc sulphate solution and methanol containing deuterium-labelled isotope internal standards. 25OHD was finally extracted with hexane, vortexed thoroughly, and then centrifuged for 10 min at 4 °C at 3148 × *g*. The upper hexane phase was then transferred into glass vials and dried under nitrogen at 40 °C for 25 min, and the dried residue was reconstituted in 150 μL methanol/water (70:30) and loaded onto the LC-MS/MS system. Chromatographic separation by LC-MS/MS was performed using a Phenomenex Kinetex PFP analytical column (100 × 3.0 mm, 2.6 μm; Phenomenex Inc. Torrance, CA, USA) with methanol as mobile phase A and 0.1% formic acid in water as mobile phase B. The isocratic gradient was as follows: 0–2.0 min, 70% A; 2.0–5.0 min, 70%–75% A; 5.0–6.5 min, 75% A; 6.5–10.0 min, 75%–80% A; 10.0–11.0 min, 80% A; 11.01–12.0 min, 90% A; and 12.01–13.0 min, 70% A. The flow rate was 0.5 mL/min. The column oven was maintained at 45 °C throughout the analysis. The deuterated analogue of 25OHD_3_ was used as an internal standard for 3-epi 25OHD_3_ and 25OHD_3_, and the deuterated analogue of 25OHD_2_ was used as an internal standard for 25OHD_2_. The MS/MS detection was operated in positive electrospray ionization mode. The multiple reaction monitoring (MRM) mode of operation was used, and the MRM transitions used for each analyte were as follows: *m/z* 413.3 → 395.3 (25OHD_2_), 401.4 → 383.4 (25OHD_3_), 416.3 → 398.3 (25OHD_2_ internal standard, [^2^H]_3_-25OHD_2_), and 404.4 → 386.4 (25OHD_3_ internal standard, [^2^H]_3_-25OHD_3_). Calibration curves were constructed by plotting the ratio of chromatography peak areas for 25OHD_2_ and 25OHD_3_ and their respective internal standards against the known concentrations, followed by linear regression to fit the data. The limits of quantification (LOQs) for 25OHD_2_ and 25OHD_3_ were 1.8 and 1.2 ng/mL, respectively. The specific level of 3-epi 25OHD_3_ was quantified using the calibration of 25OHD_3_ and [^2^H]_3_-25OHD_3_ as internal standards. A representative chromatograph is shown in Supplementary Figure 1. The linearity range for both 25OHD_3_ and 25OHD_2_ was 2.5–200 ng/mL, and both calibration curves produced a correlation coefficient higher than 0.999. Accuracy was validated by analysing the National Institute of Standards and Technology (NIST) SRM 972a. Compared with the reference values of SRM 972a, the accuracy of LC-MS/MS for measurements of 25OHD_2_, 25OHD_3_, and 3-epi 25OHD_3_ were 104.5%–106.8%, 99.5%–105.9%, and 108.0%–109.9%, respectively. Recovery was estimated by spiking serum samples with two levels of 25OHD_2_ and 25OHD_3_ and analysing in triplicate. Recovery was calculated as the ratio of the measured value and the amount of standard used to spike the sample. The mean recoveries for 25OHD_2_ and 25OHD_3_ were all near 100%. Precision was evaluated by analysing three levels of quality control samples from Bio-Rad (Liquichek^TM^ Specialty Immunoassay Control, LOT: 57440). The total coefficients of variation (CVs) for 25OHD_2_ and 25OHD_3_ were 4.34% (2.88%–7.01%) and 2.82% (2.45%–3.21%), respectively.

Immunoassay methods were performed on the Roche, Siemens, DiaSorin, Abbott, and IDS platforms, including Roche Elecsys Vitamin D Total (Lot: 171102, Instruction for Use(IFU) version: 06268668001V1,02/2011), Siemens ADVIA Centaur Vitamin D Total (Lots: 39566029, 10631021; IFU version: 10699313, 08/2012), DiaSorin Liaison XL Total Vitamin D (Lot: 131192E; IFU version: zh310600 43005, 09/2014), Abbott Architect 25-OHD Vitamin D (Lot: 02614E000; IFU version:49-8941/R02,05/2012), and IDS-iSYS (Lot:1996; IFU version: IS-2700SPL V02,05/2014), respectively.

### Statistics

Data were analysed by Passing-Bablok regression and Bland-Altman plots to evaluate comparisons between methods. Paired *t-test* was used to compare 25OHD results between methods. The cut-off for vitamin D deficiency was 20 ng/mL^1^,^18^. Agreement between methods was assessed using inter-rater agreement (Kappa values)^9^. Kappa coefficients were calculated to assess the level of agreement among different methods to identify clinically relevant hypovitaminosis (20 ng/mL). Kappa values higher than 0.6 were indicative of agreement, while values higher than 0.8 indicated excellent agreement^9^. Accuracy was expressed as the percentage of individuals with 25OHD measured by immunoassay within 15% (P_15_) or 30% (P_30_) of 25OHD measured by LC-MS/MS. The P_15_ and P_30_ values for the five immunoassays were compared with each other by McNemar’s test. Statistical analyses were performed using Microsoft Excel 2007 (Microsoft Corporation, USA) and MedCalc Statistical Software (version 13.3.3, Broekstraat, Mariakerke, Belgium).

## Results

### Performance of the automated immunoassays

While all immunoassays detected 25OHD_2_, the efficiencies varied. Only DiaSorin achieved 100% detection efficiency for detection of 25OHD_3_ (Table 1). Most immunoassays exhibited less than 3% cross-reactivity with 3-epi 25OHD_3_; however, the Roche system was less efficient at separating 3-epi 25OHD_3_ from 25OHD_3_, exhibiting a cross-reactivity of 91%. Interestingly, the Roche system had a relatively narrow analytical measurement range (3–70 ng/mL). In contrast, the Roche and DiaSorin systems had relatively better precision than the other platforms, with both CVs and inter-CVs of less than 5%.

### Comparisons of methods

**Figure 1:**
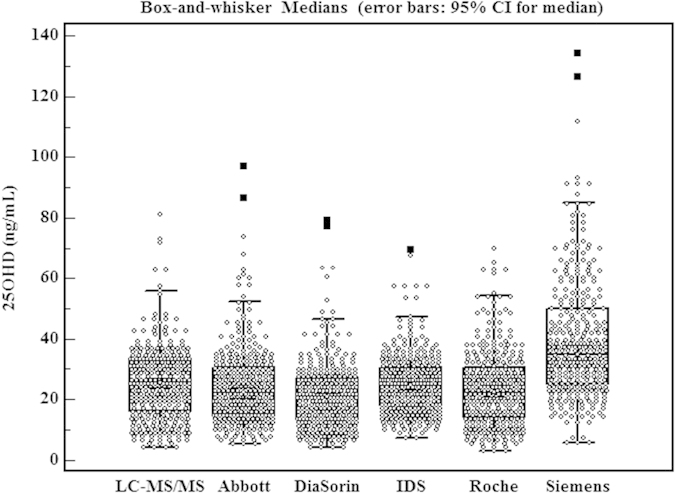
Box-and-whisker plot showing the distribution of results for all assays tested in a total of 332 serum samples. The central boxes represent the 25th to 75th percentile range. The lines inside the boxes show the median value and 95% CI for the median value for each method tested. The whiskers extend from the minimum to the maximum value, excluding outliers. An outlier value is defined as a value that exceeds the upper or lower quartile plus or minus 1.5 times the interquartile range.

Of the total samples, the mean ± SD 25OHD was as follows (Fig. 1): 25.5 ± 12.0 ng/mL (LC-MS/MS), 24.6 ± 12.7 ng/mL (Abbott), 21.7 ± 11.1 ng/mL (DiaSorin), 25.4 ± 9.9 ng/mL (IDS), 23.9 ± 12.5 ng/mL (Roche), and 39.5 ± 19.8 ng/mL (Siemens). Paired *t-test* showed the result of the Siemens system was significantly higher than the LC-MS/MS result (P < 0.05)., while the results of DiaSorin, Roche, and Abbott immunoassays were lower than the LC-MS/MS results (Paired T tests, P < 0.05). Accuracy, as estimated by P_15_ and P_30,_ showed that the Abbott, DiaSorin, IDS, and Roche systems did not differ significantly from each other (P_15_: 43.67%, 49.70%, 45.18%, and 48.80%, respectively, McNemar’s test, *p* > 0.05; P_30_: 76.20%, 81.93%, 76.81%, and 75.60%, respectively, McNemar’s test, *p* > 0.05), but their accuracy values were significantly higher than that of the Siemens system (P_15_: 14.76%; P_30_: 27.11%, McNemar’s test, *p* < 0.01).

Most immunoassays (except for Siemens) showed acceptable diagnostic agreement with LC-MS/MS (Kappa > 0.6), while Roche had the best Kappa value (Table 2). When there was no detectable 25OHD_2_ or 3-epi 25OHD_3_, all immunoassays, except for Siemens, were in excellent agreement. When males and females were analysed separately, the 25OHD immunoassay results produced a trend similar to that observed in the total sample (Supplemental Table 1).

Moreover, considering the differences between the methods, we used regression models (calculated from the total samples) to transfer the cut-offs for immunoassay methods. After transferring, the cut-offs for the definition of hypovitaminosis D were 20 (Abbott), 17 (DiaSorin), 21 (IDS), 19 (Roche), and 31 ng/mL (Siemens), respectively. Using these transferred cut-offs, Siemens showed the biggest improvement in Kappa value (increased from 0.410 to 0.637), and most of the immunoassays showed an acceptable and improved agreement with LC-MS/MS (Table 2).

### Effects of 25OHD_3_ analogues on quantification methods

**Figure 2:**
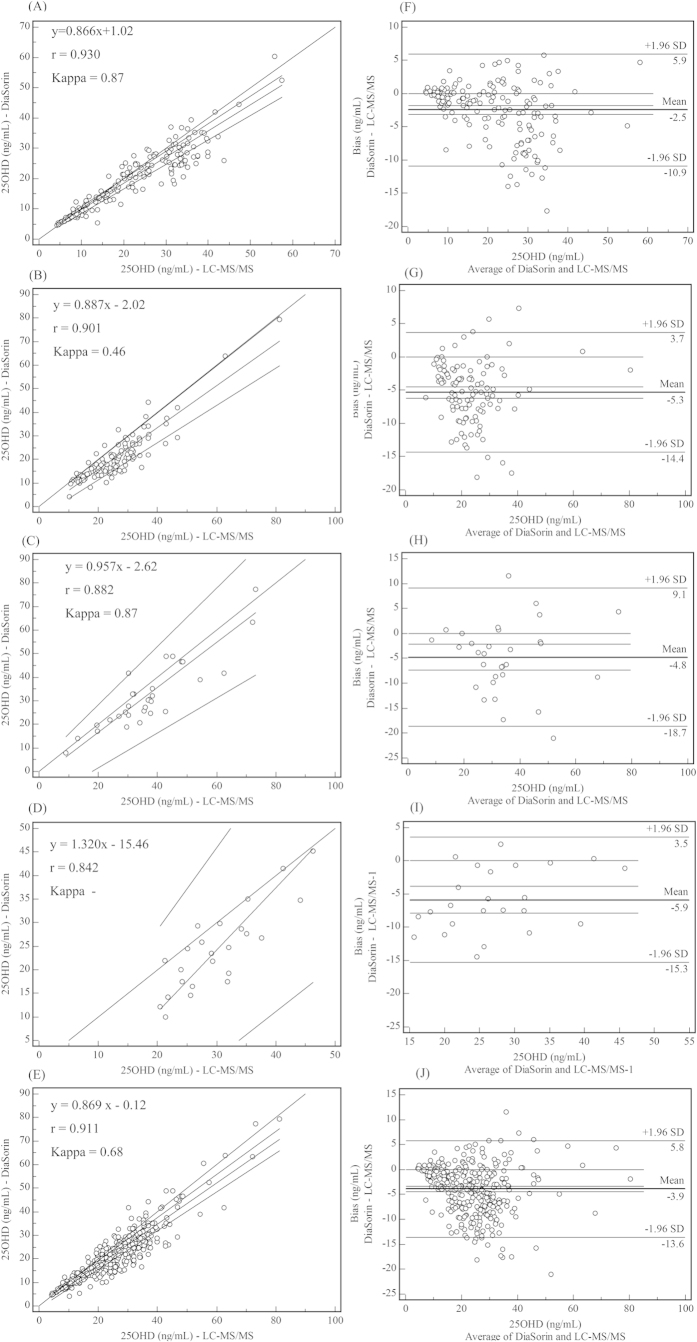
Comparison of DiaSorin and LC-MS/MS. A–E are Passing-Bablok regression analyses for 166 serum samples containing only 25OHD_3_, 111 serum samples containing both 25OHD_3_ and 25OHD_2_, 31 serum samples containing 25OHD_3_, and all 332 serum samples, respectively. F–J are Bland-Altman plots showing the bias between DiaSorin and LC-MS/MS for 166 serum samples containing only 25OHD_3_; 111 serum samples containing both 25OHD_3_ and 25OHD_2_; 31 serum samples containing 25OHD_3_; 24 samples containing 25OHD_3_, 25OHD_2_, and 3-epi 25OHD_3_; and all 332 serum samples, respectively.

Although the DiaSorin system was supposed to detect 25OHD with 100% efficiency and specificity for 25OHD_3_, the correlation coefficient declined, the bias increased, and the Kappa value decreased significantly when the samples contained 25OHD_2_ or 3-epi 25OHD_3_ (Fig. 2). The other four immunoassays exhibited a similar trend (Table 2). The level of 25OHD_2_ correlated significantly with the bias between immunoassay methods (for Abbott, DiaSorin, IDS, Roche, and Siemens: r = 0.848, 0.909, 0.834, 0.849, and 0.282, respectively) and LC-MS/MS (Supplemental Figure 2). And it was shown that when there was no 25OHD_2_, bias% at the medical decision level of Roche was the smallest, however, when 25OHD2 present, bias% at medical decision level significantly increased(Supplemental Table 2).

### Effects of the vacuum blood collection tubes on quantification methods

**Figure 3:**
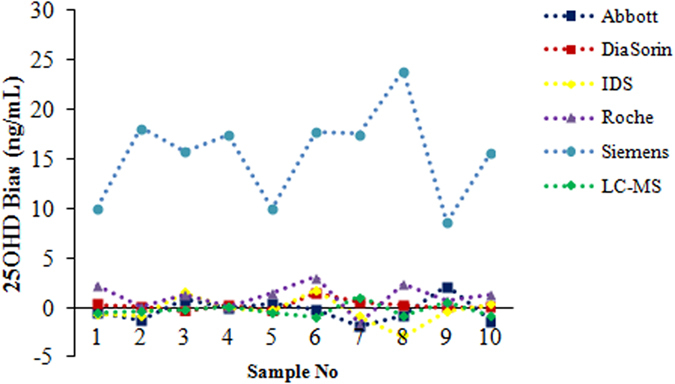
Bias between 25OHD results in VACUETTE 4-mL tubes with gel and clot activator and in VACUETTE 4-mL additive-free tubes of each method. X-axis: sample numbers; Y-axis: 25OHD results in VACUETTE 4-mL tubes with gel and clot activator minus 25OHD results in VACUETTE 4-mL additive-free tubes.

**Figure 4:**
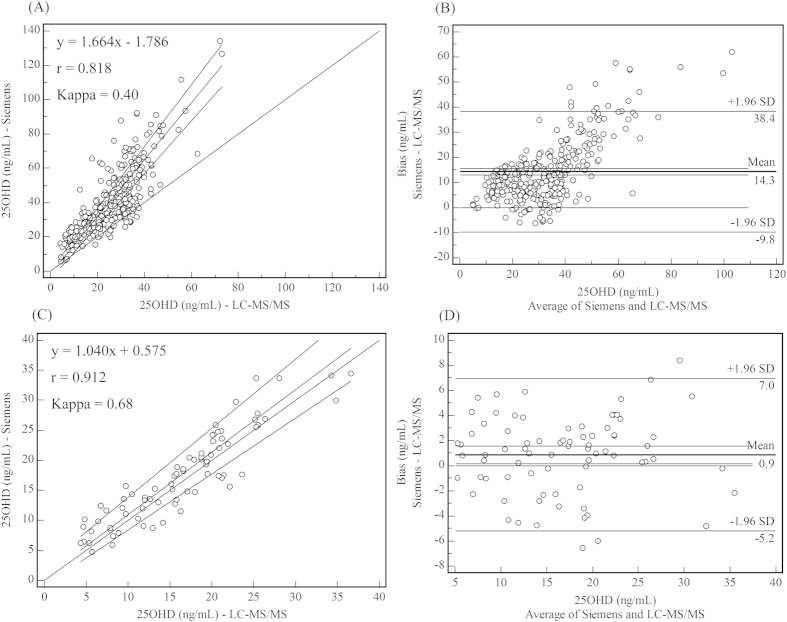
Comparison of 25OHD results for Siemens and LC-MS/MS in VACUETTE 4-mL tubes with gel and clot activator and VACUETTE 4-mL no-additive -free tubes. A and B are Passing-Bablok regression and Bland-Altman plots of 332 25OHD samples analysed using Siemens and LC-MS/MS for blood collected in VACUETTE 4-mL tubes with gel and clot activator; C and D are Passing-Bablok regression and Bland-Altman plots of 77 25OHD samples analysed using Siemens and LC-MS/MS for blood collected in VACUETTE 4-mL additive-free tubes.

Next, to clarify whether the vacuum blood collection tubes affected the accuracy of immunoassays, we analysed 10 samples after blood collection into both VACUETTE 4-mL additive-free tubes and VACUETTE 4-mL tubes with gel and clot activator, and analyzed all samples by the five immunoassays and LC-MS/MS. Results was shown as Fig. 3, and it was shown that samples collected in VACUETTE 4-mL tubes with gel and clot activator exhibited apparently higher values than samples in tubes with no additives (mean bias (SD): 12.7 (4.3) ng/mL, P < 0.01) using the Siemens system. An additional 67 volunteers were recruited and their serum was collected in VACUETTE 4-mL additive-free tubes, and for the total 77 samples collected in VACUETTE 4-mL additive-free tubes were all analyzed both by Siemens system and LC-MS/MS, and the correlation coefficient between the two methods improved, bias decreased significantly, and with a slope close to 1, indicated agreement (Kappa = 0.68) (Fig. 4).

## Discussion

In this study, we examined differences in the accuracies of five different immunoassay systems for analysis of vitamin D and vitamin D analogues in blood samples from 332 individuals. Our data demonstrated that most of the systems, with the exception of the Siemens system, exhibited good acceptability and accuracy. Moreover, with the Siemens system, the use of particular blood collection tubes affected the results substantially, and we summarized the effects of 25OHD analogues and VACCUTTE tubes to immunoassays in Supplemental Table 3. These data have implications in the further development and application of assays to measure vitamin D levels.

In recent years, various organizations have carried out vitamin D standardization studies, such as the US Centers for Disease Control and Prevention’s Vitamin D Standardization-Certification Program (CDC VDSCP), the international Vitamin D External Quality Assessment Scheme (DEQAS), and the Vitamin D Standardization Program (VDSP) established in 2010 by the National Institutes of Health (NIH) Office of Dietary Supplements, US Centers for Disease Control and Prevention, US National Institute of Standards and Technology (NIST), and the Belgium Laboratory for Analytical Chemistry (Ghent, Belgium)^19^,^20^. These organizations have also promoted the improvement of vitamin D products to achieve efficient detection of 25OHD_2_ and 25OHD_3_, as well as 3-epi 25OHD_3_. However, to the best of our knowledge, few studies have focused on the effects of 25OHD_2_ and 3-epi 25OHD_3_ on immunoassay methods. Therefore, our current study was the first to compare the effects of both 25OHD_2_ and 3-epi 25OHD_3_ on the precision and accuracy of various immunoassays.

In recent years, researchers have compared different methods for detection of 25OHD; however, while high correlations between methods were found in some studies, it was not possible to consistently determine the best method. Farrell *et al.*^8^ reported that all immunoassay methods tested were highly correlated with LC-MS/MS, with a regression coefficient above 0.9, but the Roche system produced the poorest correlation coefficient (r = 0.679); indeed, the assay used in the previous study was only able to detect 25OHD_3_. However, with a focus on vitamin D_2_, Roche has improved their products for detection of both 25OHD_2_ and 25OHD_3_ and we used the improved assay in our experiment. In a previous report^21^, Siemens, DiaSorin, and Roche were shown to produce similar and acceptable correlations with LC-MS/MS, while Koivula *et al.*^22^ reported that the Abbott, DiaSorin, IDS, and Siemens systems produced poor regression coefficients, and only the Siemens and IDS systems were in good clinical agreement with LC-MS/MS. However, Ajuria-Morentin *et al.*^9^ showed that the Siemens system produced the poorest correlation and largest bias in comparison to the other immunoassay methods, consistent with our results.

Substantial differences among immunoassay methods may be related, in part, to their different capacities for the measurement of 25OHD_2_ and 3-epi 25OHD_3_. Although all of the manufacturers claimed that their immunoassay methods could detect 25OHD_2_, with DiaSorin claiming that their antibody had equal molar efficiency for 25OHD_2_ and 25OHD_3_, all regression coefficients decreased when samples contained 25OHD_2_, and the bias between immunoassay methods and LC-MS/MS correlated significantly with the level of 25OHD_2_. Currently, both vitamin D_2_ and vitamin D_3_ supplements are used in China and the USA, which is problematic for diagnosing vitamin D deficiency since our results indicate that the consistency of 25OHD_2_ measurement by common immunoassays might not be satisfactory. These results were inconsistent with the results of a study by Le Goff *et al.*, who showed that only the Abbott and Siemens systems produced unsatisfactory reactivity with 25OHD_2_^23^. Moreover, while all manufacturers (except Roche) claimed that their assays had little cross-reactivity with 3-epi 25OHD_3_, our results showed that for all immunoassays tested, the correlation with LC-MS/MS decreased significantly when samples contained 3-epi 25OHD_3._ The levels of 3-epi 25OHD_3_ in our samples were relatively low relative to the total 25OHD. On one hand, this may support the notion that 3-epi 25OHD_3_ would not be expected to routinely affect LC-MS/MS methods that could not distinguish and separate 3-epi 25OHD_3_ from 25OHD_3_^24^. On the other hand, given the low levels of 3-epi 25OHD_3_ present in samples, it had a relatively significant effect on the results of the immunoassay. Thus, the low levels and infrequency of 3-epi 25OHD_3_ in the samples in our study represent an important limitation of our work. Future studies should examine the effects of increased concentrations of 3-epi 25OHD_3_ in samples.

Previous studies have shown that it is necessary for laboratories to develop site-specific reference intervals and protocols for achieving consistent results^9^. Additionally, our results support the proposal that site-specific reference intervals are necessary to improve uniformity; however, the degree of improvement was platform-dependent. For example, the Siemens systems improved more than the other methods when the cut-off values were transferred according to the regression equation.

The cut-off for vitamin D deficiency has been a controversial topic^25^. IOM recommends that 12 ng/mL can satisfy the necessary requirements for normal adults^26^. In contrast, endocrinologists reviewed many studies on vitamin D and concluded that 20 ng/mL was a better cut-off for the definition of vitamin D deficiency^18^. According to our results, the controversy may be exacerbated by the different immunoassay methods used in various studies. In the future, it will be necessary to define vitamin D deficiency based on papers that use a reference method, such as LC-MS/MS. However, considering the great bias produced by Siemens, detected in the current study and by others^9^, this particular Siemens system is expected to produce variable results in the presence of vitamin D analogues and is sensitive to the cut-off value used. Siemens had passed the First Hormone Standardization Program for Vitamin D organized by the US CDC in October of 2014. The standardization program allows a mean bias within 5% as acceptance criteria, and Siemens Healthcare Diagnostics, along with IDS, are the only certified immunoassay methods. Therefore, the results of our study and the study by Ajuria-Morentin *et al.*^9^ are somewhat confusing in this context. Although Borai reported that BD serum separator tubes did not affect the Abbott and DiaSorin immunoassays in measurement of 25OHD_3_^27^, the effects of VACUETTE blood collection tubes on Siemens immunoassay were unclear. In our analysis, we found that the use of a VACUETTE tube with gel and clot activator, which is commonly used in our hospital to measure 25OHD, resulted in significantly higher measurements than samples collected in tubes with no additive using the Siemens system. Importantly, however, LC-MS/MS did not produce a significant bias due to the type of tube used. 25OHD results for blood samples collected in VACUETTE tubes with no additives showed excellent performance in comparison to LC-MS/MS. The reasons for these observations are not understood. It is possible that the clot activator, separating gel, or some other elements in the VACUETTE 4-mL tube with gel and clot activator increased the nonspecific cross-reactivity between magnetic particle-labelled antibodies and acridinium ester, leading to increased chemiluminescence values. These possibilities cannot be fully elucidated since the tube manufacturers and Siemens maintain confidentiality with respect to the specific compositions of their products. Therefore, although the specific effects are not known, particularly with the Siemens system, it will be important to determine which blood collection tubes are appropriate for reliable use. Our results highlight the necessity of evaluating the effects of the blood collection tubes when choosing 25OHD immunoassays. Further studies are needed to clarify the mechanisms through which the blood collection tube interferes with measurement of 25OHD using the Siemens system.

In summary, our results showed that most automated immunoassays had acceptable correlation and agreement with LC-MS/MS when there was no detectable 25OHD_2_ or 3-epi 25OHD_3_. However, the presence of either 25OHD_2_ or 3-epi 25OHD_3_ had substantial effects on the results of immunoassay methods. Therefore, when defining the cut-off value for vitamin D deficiency, the difference between methods should be considered. Moreover, when using the Siemens system, it is essential to use appropriate vacuum blood collection tubes to measure 25OHD in clinical laboratories or for epidemiological investigations.

## Additional Information

**How to cite this article**: Yu, S. *et al.* 25OHD analogues and vacuum blood collection tubes dramatically affect the accuracy of automated immunoassays. *Sci. Rep.* **5**, 14636; doi: 10.1038/srep14636 (2015).

## Supplementary Material

Supplementary Information

## Figures and Tables

**Table 1:** Performance of the automated immunoassays. ^a^The data was collected from the Instruction for Use of respective manufactures.

	**Abbott**	**Siemens**	**Roche**	**IDS**	**DiaSorin**
Range of detection^a^ (ng/mL)	8.0–160.0	4.2–150.0	3–70	5.5–140	4–150
LOD^a^ (ng/mL)	3.1	3.2	3	—	—
LOQ^a^ (ng/mL)	8	4.2	5	5.5	4
Cross-reactivity^a^					
25OHD_3_ (%)	105	100.7	100	102	100
25OHD_2_ (%)	82	104.5	92	95	100
3-epi 25OHD_3_ (%)	2.7	1.1	91	1	1.3
Serum Pool					
Pool 1 (ng/mL)	8.6 ± 0.9	13.2 ± 1.7	6.7 ± 0.4	9.4 ± 1.1	6.5 ± 0.4
Pool 2 (ng/mL)	17.6 ± 0.9	23.4 ± 1.4	16.8 ± 0.5	21.5 ± 1.4	15.6 ± 0.5
Pool 3 (ng/mL)	25.8 ± 1.0	33.5 ± 3.2	26.7 ± 0.9	28.6 ± 0.9	21.6 ± 0.6
CV: between runs (%)	4.4 (3.1–6.6)	10.5 (6.4–15.9)	3.6 (2.6–5.4)	5.9 (3.0–9.3)	3.3 (2.1–5.7)
CV: total (%)	6.9 (3.6–11.8)	9.9 (6.1–14.1)	4.0 (2.7–6.0)	7.1 (3.3–11.6)	3.7 (2.6–5.3)

**Table 2:** Comparisons between LC-MS/MS methods and automated immunoassays.

	**Slope**	**95% CI**	**Intercept**	**95% CI**	**r**	**95% CI**	**Mean Bias (SD) (ng/mL)**	**Kappa**^b^
Total samples (332)								
Abbott	0.934	0.873–1.002	1.1	−0.39–2.2	0.859	0.828–0.885	−1.0(6.6)	0.66(0.66)
DiaSorin	0.869	0.822–0.916	−0.12	−1.10–0.85	0.911	0.891–0.928	−3.9(4.9)	0.68(0.76)
IDS	0.791	0.744–0.840	5.23	4.27–6.39	0.871	0.842–0.895	−0.2(5.9)	0.69(0.67)
Roche	1.034	0.978–1.090	−2.15	−3.37– −1.04	0.882	0.855–0.904	−1.7(6.0)	0.74(0.76)
Siemens	1.664	1.536–1.797	−1.79	−4.74–0.77	0.818	0.779–0.851	14.3(12.3)	0.40(0.63)
Siemens^a^	1.04	0.934~1.153	0.58	−0.97~ 2.25	0.912	0.865~0.943	0.9(3.1)	0.68(0.82)
Only 25OHD_3_ (166)								
Abbott	0.997	0.927–1.061	1.28	0.16–2.63	0.911	0.881–0.934	1.1(5.0)	0.84(0.85)
DiaSorin	0.866	0.814–0.912	1.02	0.22–1.56	0.93	0.906–0.948	−2.5(4.3)	0.87(0.90)
IDS	0.768	0.713–0.823	7.46	6.29–8.43	0.898	0.864–0.924	2.0(5.3)	0.80(0.84)
Roche	0.992	0.931–1.056	0.09	−0.92–1.14	0.911	0.881–0.934	−0.4(4.9)	0.90(0.90)
Siemens	1.483	1.359–1.629	2.02	−0.54–4.54	0.872	0.829–0.904	12.5(9.6)	0.47(0.72)
Both 25OHD_2_ and 25OHD_3_ (111)								
Abbott	0.93	0.649–0.826	3.23	0.53–5.06	0.864	0.807–0.904	−4.3(5.3)	0.44(0.47)
DiaSorin	0.887	0.792–0.995	−2.02	−4.69– −0.04	0.901	0.859–0.931	−5.3(4.6)	0.46(0.58)
IDS	0.835	0.745–0.935	1.44	−1.27– 3.78	0.861	0.803–0.902	−2.4(5.3)	0.58(0.47)
Roche	1.075	0.948–1.203	−6.25	−9.04– −3.03	0.859	0.801–0.901	−4.0(5.6)	0.55(0.61)
Siemens	1.997	1.552–2.457	−11.51	−21.94– −0.81	0.599	0.459–0.710	14.2(13.5)	0.16(0.42)
25OHD_3_ and 3-epi 25OHD_3_(31)								
Abbott	1.365	1.024–1.707	−11.84	−26.89– −1.53	0.865	0.736–0.933	−0.9(10.4)	0.76(0.76)
DiaSorin	0.957	0.740–1.240	−2.62	−13.24 – 3.51	0.882	0.768–0.942	−4.8(7.1)	0.87(0.84)
IDS	0.879	0.766–1.096	3.65	−5.43–7.16	0.853	0.715–0.927	−1.2(7.7)	0.52(0.52)
Roche	1.071	0.880–1.333	−1.74	−11.0–5.12	0.873	0.752–0.938	−3.8(7.6)	0.64(0.38)
Siemens	2.051	1.600–2.832	−14.02	−42.21–2.54	0.811	0.637–0.906	22.6(17.8)	− c(0.52)
25OHD_2_, 25OHD_3_ and 3-epi 25OHD_3_ (24)								
Abbott	1.593	1.029–2.433	−19.9	−41.89– −5.83	0.659	0.349–0.840	−2.1(8.7)	—
DiaSorin	1.32	0.985–1.751	−15.46	−28.24– −6.63	0.842	0.664–0.930	−5.9(4.8)	—
IDS	1.012	0.678–1.658	−4.13	−21.96–6.38	0.744	0.487–0.883	−3.7(5.1)	—
Roche	1.714	1.198–2.609	−22.63	−51.23– −9.83	0.76	0.515–0.891	−2.5(9.0)	—
Siemens	2.51	1.902–3.494	−29.37	−59.86– −11.35	0.858	0.695–0.937	16.7(11.4)	—

^a^The results were calculated from the 77 samples collected in the VACCUTTE tubes with no additive.

^b^The Kappa values in the brackets were calculated using the respective transferred cut-offs for each method.

^c^The number of Siemens was classified as vitamin D deficiency (<20 ng/mL) was zero leading to Kappa value = 0.
