# Supplementary material for: 25OHD analogues and vacuum blood collection tubes dramatically affect the accuracy of automated immunoassays
Source: Sci Rep. 2015 Sep 30;5:14636. doi: 10.1038/srep14636 (PMC4588576; doi:10.1038/srep14636)

**Supplementary data for**

**25OHD analogues and vacuum blood collection tubes dramatically affect the accuracy of automated immunoassays**

Songlin Yu^1＋^, Xinqi Cheng^1＋^, Huiling Fang^1＋^, Ruiping Zhang^2^, Jianhua Han^1^, Xuzhen Qin^1^, Qian Cheng^1^, Wei Su^1^, Li’an Hou^1^, Liangyu Xia^1^, Ling Qiu *

^1^Department of Clinical Laboratory, Peking Union Medical College Hospital, Chinese Academy of Medical Sciences, Beijing 100730, China

^2^Department of Clinical Laboratory, China-Japan Hospital, Beijing 100029, China

| **Supplemental Table 1. Comparisons between LC-MS/MS methods and automated immunoassays in males and females** | | | | | | | | | | | | | | | |
| --- | --- | --- | --- | --- | --- | --- | --- | --- | --- | --- | --- | --- | --- | --- | --- |
|  | Slope | 95% CI | Intercept | | 95% CI | | r | | 95% CI | | Bias (SD) | | 95% CI | | Kappa |
| Male (106) | |  |  | |  | |  | |  | |  | |  | |  |
| Abbott | 0.904 | 0.8023–1.047 | 1.12 | | -2.10–3.39 | | 0.885 | | 0.836–0.921 | | -1.6(6.5) | | -2.9 – -0.35 | | 0.68(0.67) |
| DiaSorin | 0.88 | 0.815-0.940 | 0.33 | | -1.2–1.15 | | 0.932 | | 0.901–0.953 | | -4.1 (5.0) | | -3.1–-5.1 | | 0.78 (0.83) |
| IDS | 0.767 | 0.699–0.843 | 5.99 | | 4.26–7.74 | | 0.882 | | 0.832–0.919 | | -0.4 (6.6) | | -1.7–0.9 | | 0.66 (0.65) |
| Roche | 1.099 | 0.996–1.233 | -4.03 | | -7.23–-1.44 | | 0.872 | | 0.817–0.911 | | -1.2 (7.2) | | -2.6–0.2 | | 0.85 (0.85) |
| Siemens | 1.671 | 1.476–1.908 | 0.63 | | -4.71–5.59 | | 0.596 | | 0.454–0.708 | | 17.8 (12.5) | | 15.4–20.3 | | 0.14 (0.71) |
| Female (226) | | |  | |  | |  | |  | |  | |  | |  |
| Abbott | 0.897 | 0.823–0.975 | 1.6 | | 0.03–2.7 | | 0.853 | | 0.813–0.885 | | -1.5 (6.6) | | -2.4–-0.7 | | 0.63 (0.68) |
| DiaSorin | 0.805 | 0.753–0.865 | 0.61 | | -0.61–1.61 | | 0.894 | | 0.864–0.917 | | -4.7 (5.4) | | -5.4– -4.0 | | 0.62 (0.74) |
| IDS | 0.748 | 0.692–0.808 | 5.31 | | 4.01–6.60 | | 0.861 | | 0.823–0.891 | | -0.9 (6.2) | | -1.8– -0.1 | | 0.71 (0.68) |
| Roche | 0.942 | 0.881–1.008 | -0.76 | | -2.40– 0.54 | | 0.893 | | 0.864–0.917 | | -2.8 (5.5) | | -3.5–-2.1 | | 0.69 (0.73) |
| Siemens | 1.475 | 1.340–1.623 | -0.52 | | -3.20–2.53 | | 0.835 | | 0.790–0.871 | | 11.8 (11.3) | | 10.3–13.3 | | 0.52 (0.61) |
| a. The Kappa values in the brackets were calculated using the respective transfer cut-offs of each method. | | | | | | | | | | | | | | | |
| Supplemental Table 2 Bias% in the Medical decision level | | | | | | | | | | | | | |  | |
| Medical decision level (ng/mL) | | | | Bias% | | | | | | | | | |  | |
|  |  |  |  | 12 | | 20 | | 30 | | 100 | | 150 | |  | |
| Total samples (332) | | | |  | |  | |  | |  | |  | |  | |
| Abbott | | | | 2.57% | | -1.10% | | -2.93% | | -5.74% | | -5.87% | |  | |
| DiaSorin | | | | -15.00% | | -14.60% | | -14.40% | | -13.09% | | -14.08% | |  | |
| IDS | | | | 22.68% | | 5.25% | | -3.47% | | -20.03% | | -17.41% | |  | |
| Roche | | | | -14.52% | | -7.35% | | -3.77% | | 4.28% | | 1.97% | |  | |
| Siemens | | | | 51.48% | | 57.45% | | 60.43% | | 67.22% | | 65.21% | |  | |
| Siemens^a^ | | | | 8.83% | | 6.90% | | 5.93% | | 4.91% | | 4.39% | |  | |
| Only 25OHD_3_ (166) | | | |  | |  | |  | |  | |  | |  | |
| Abbott | | | | 10.32% | | 6.05% | | 3.92% | | 0.56% | | 0.50% | |  | |
| DiaSorin | | | | -4.87% | | -8.27% | | -9.97% | | -12.43% | | -12.68% | |  | |
| IDS | | | | 38.90% | | 14.04% | | 1.61% | | -22.35% | | -18.28% | |  | |
| Roche | | | | -0.07% | | -0.37% | | -0.52% | | 0.09% | | -0.76% | |  | |
| Siemens | | | | 65.16% | | 58.43% | | 55.06% | | 49.19% | | 49.67% | |  | |
| Both 25OHD_2_ and 25OHD_3_ (111) | | | | | |  | |  | |  | |  | |  | |
| Abbott | | | | 19.85% | | 9.10% | | 3.72% | | -6.17% | | -4.88% | |  | |
| DiaSorin | | | | -28.11% | | -21.39% | | -18.03% | | -10.41% | | -12.65% | |  | |
| IDS | | | | -4.55% | | -9.34% | | -11.74% | | -15.67% | | -15.57% | |  | |
| Roche | | | | -44.56% | | -23.73% | | -13.31% | | 8.38% | | 3.35% | |  | |
| Siemens | | | | 3.80% | | 42.18% | | 61.37% | | 100.34% | | 92.07% | |  | |
| a. The results were calculated from the 77 samples collected in the VACCUTTE tubes with no additive. | | | | | | | | | | | | | |  | |

| Supplemental Table 3. Effects of 25OHD analogues and VACCUTTE tubes to immunoassays | | | |
| --- | --- | --- | --- |
|  | 25OHD_2_ | 3-epi 25OHD_3_ | VACCUTTE tubes with gel and clot activator |
| Abbott | + | + | - |
| DiaSorin | + | + | - |
| IDS | + | + | - |
| Roche | + | + | - |
| Siemens | + | + | + |
| *+positive,- negetive | | | |

Supplemental figure 1


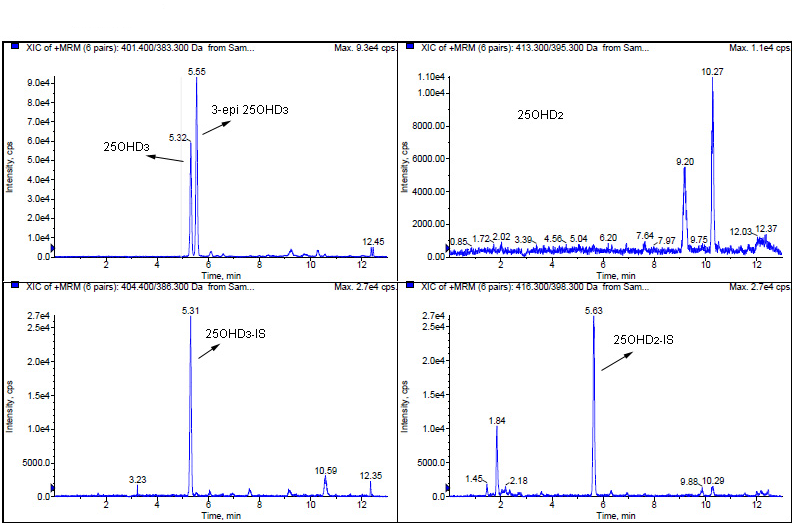


Supplemental Figure 2


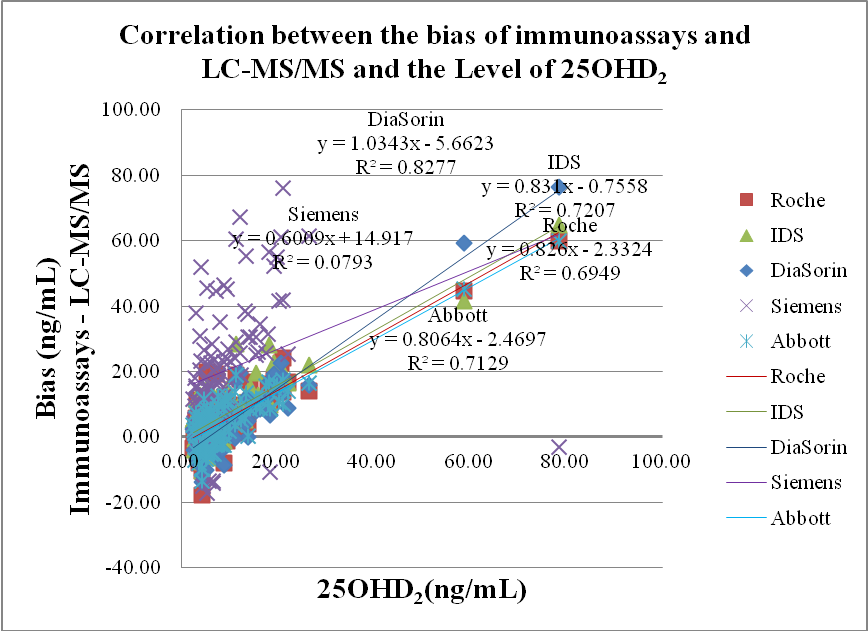

Supplement: Supplementary Information [file srep14636-s1.doc]
